# Association between healthiness of community food environments and diet-related health outcomes in regional Australia: an ecological study

**DOI:** 10.1186/s12889-026-26663-3

**Published:** 2026-02-17

**Authors:** Alemayehu Digssie Gebremariam, Katherine Kent, Chris Brennan-Horley, Karen Charlton

**Affiliations:** 1 School of Medical, Indigenous and Health Sciences, Faculty of Science, Medicine and Health, University of Wollongong, Wollongong, NSW Australia; 2 Department of Public Health, College of Health Sciences, Debre Tabor University, Debre Tabor, Ethiopia; 3 School of Social Sciences, Faculty of Arts, Social Sciences and Humanities, University of Wollongong, Wollongong, NSW Australia; 4 School of Health Sciences, University of Newcastle, Newcastle, NSW 2308 Australia; 5 Hunter Medical Research Institute, Newcastle, NSW 2308 Australia

**Keywords:** Community food environment, Diet-related health, Diabetes, Regional australia, Ecological study

## Abstract

**Background:**

The community food environment influences access to healthy and affordable foods and is considered a risk factor for diet-related health outcomes. An ecological approach helps identify links between food environments and health outcomes by generating hypotheses. Evidence is limited and inconsistent, particularly in regional Australia. This study examined the association between community food environment metrics and diet-related health outcomes in a large regional area of Australia.

**Methods:**

An ecological analysis was conducted using secondary data from the 2021 census at the Statistical Area Level 2 (SA2) and from the Public Health Information Development Unit (PHIDU), according to Population Health Area (PHA) levels. Health outcomes included the age-standardised prevalence of diet-related health outcomes (e.g., diabetes, kidney disease, overweight) and self-rated health. Food environment metrics, calculated at both SA2 and PHA levels included: (1) the ratio of unhealthy to healthy/less healthy food outlets; (2) the Modified Retail Food Environment Index (mRFEI) - the proportion of healthy food outlets among all available outlets; (3) the Relative Healthy Food Availability (RHFA) - the proportion of supermarkets and greengrocers relative to fast-food outlets; and (4) the Relative Fast Food Availability (RFFA) - the proportion of fast-food outlets among all available outlets. Spearman’s correlation was used to examine associations.

**Results:**

A higher ratio of unhealthy to healthy/less healthy food outlets was positively associated with diabetes (ρ = 0.48, *P* = 0.001), kidney disease (ρ = 0.42, *P* = 0.006), stroke (ρ = 0.32, *P* = 0.042), and poor/fair self-rated health (ρ = 0.41, *P* = 0.029). A higher RFFA was positively associated with diabetes (ρ = 0.6, *P* < 0.001) and stroke (ρ = 0.43, *P* = 0.005). The RHFA was negatively associated with diabetes (ρ = -0.38, *P* = 0.012) and overweight (ρ = -0.51, *P* = 0.005), while the mRFEI was negatively associated with overweight (ρ = -0.46, *P* = 0.013).

**Conclusion:**

Community food environment metrics are significantly associated with population-level diet-related health outcomes in regional Australia. These ecological findings identify key metrics for future individual-level research and inform local and state policies aimed at reducing unhealthy food outlets and increasing healthy options.

## Introduction

Diet-related noncommunicable diseases, such as cardiovascular diseases, diabetes, and obesity, are leading public health concerns in Australia and other high-income countries [1]. According to the 2024 Australian Burden of Disease Study (ABDS), cardiovascular disease alone accounts for 12% of the total disease burden [2]. An unhealthy diet, characterised by high intakes of sugar, salt, and unhealthy fats and low intakes of fruits and vegetables, is a major modifiable risk factor for noncommunicable diseases [3]. Australians living in regional, rural, and socioeconomically disadvantaged areas are disproportionately affected by diet-related health conditions [2]. In these communities, the food environment may play a significant role in barriers to healthy eating, including limited access to supermarkets, higher food prices, and fewer fresh food options due to supply chain constraints and distance [4, 5]. Public transportation is often lacking, and food environments are frequently dominated by convenience stores and fast-food outlets offering mostly unhealthy options [6]. These structural and geographic challenges may contribute to poorer diet quality and disparities in diet-related health outcomes [7]. Despite the high prevalence of diet-related health outcomes in rural Australia and the potential for dietary interventions to reduce these disparities, few interventions to improve the healthiness of the food environment have been implemented to date [8].

The association between the community food environment and diet, weight status, and health outcomes has been the topic of a number of reviews that have generally reported mostly null or inconclusive findings [9–14]. However, recent reviews by Pineda et al. [15] and Gebremariam, Kent, and Charlton [16] have reported that a dominance of unhealthy food outlets in community food environments, such as fast-food outlets and convenience stores, is associated with adverse health outcomes, including obesity, diabetes, and cardiovascular diseases. Conversely, the presence of healthy and less healthy outlets, including supermarkets, full-service restaurants, and farmers’ markets, was protective. In the Australian context, two reviews have been conducted, but they reported null findings due to the limited number of studies, particularly those focusing on health outcomes rather than just overweight and obesity. This suggests that further primary studies are needed in diverse Australian communities [13, 14].

The South Coast of New South Wales (NSW) is broadly defined as the coastal strip area south of Sydney, extending down to the Victorian border. There are four geographic regions, namely the Illawarra, Shoalhaven, Eurobodalla, and the Sapphire Coast, with an estimated 2024 population of 514, 885 people [17]. The region encompasses a mix of urban, regional, and rural areas spanning Modified Monash categories MM1 (metropolitan areas), MM2 (regional centres), through to MM5 (small rural towns) [18]. Socioeconomically, this region features a diverse range of communities, with some areas experiencing greater disadvantage compared to other parts of NSW. For example, the largest local council area of the Illawarra (Wollongong) includes suburbs among the most disadvantaged in the state, namely Port Kembla and its nearby suburbs of Cringila and Warrawong, being in the bottom two Index of Relative Socioeconomic Advantage and Disadvantage (IRSAD) deciles nationally [19]. The area is also home to heavy industry, including one of Australia’s largest steelworks, which has greatly influenced the socioeconomic landscape. Contributing factors to socioeconomic disadvantage across the region may include high unemployment rates resulting from downsizing and automation in the steel and coal industries, a high percentage of low-income households, and a significant proportion of culturally and linguistically diverse (CALD) households.

The South Coast is also affected by disparities in access to healthy food and a high prevalence of diet-related health outcomes. A study mapping the community food environment across the Illawarra and Shoalhaven regions, for example, identified a predominance of unhealthy food outlets, which was related to the level of socioeconomic disadvantage across communities [20]. Similarly, variations in the distribution of cardiometabolic risk factors have been noted across geographical areas according to socioeconomic positions [21]. Despite these indications of place-based inequities, there is limited evidence on how community food environment metrics relate to population-level diet-related health outcomes in the region. An ecological approach is a critical first step to identify which food environment metrics are most relevant and which health outcomes warrant further investigation, providing a foundation for the design of individual-level and longitudinal studies. Addressing these challenges will require interventions that go beyond the individual, to those that are supported by policy and planning initiatives to influence the community food environment, to improve the availability, accessibility, affordability, and promotion of healthy food [22]. In this context, the Food Environment Policy Index (Food-EPI) developed by the INFOMAS network provides a structured framework in which to examine which food environment domains are addressed in public policies, to identify strengths and gaps in regulatory frameworks, and to support the development of actionable policy recommendations to strengthen food environment policy [23]. Evidence from ecological studies such as this one can help inform and prioritise policy actions aligned with the framework. Thus, this study aims to explore the association between metrics of the community food environment and the prevalence of diet-related health outcomes at the Statistical Area Level 2 (SA2) and Population Health Area (PHA) levels in the South Coast of NSW.

## Methods

### Study context

The study was conducted in the predominantly regional area of the South Coast of New South Wales that comprises six Local Government Areas (LGAs): Wollongong, Shellharbour, Kiama, Shoalhaven, Eurobodalla, and Bega Valley Shire. In these local government areas, there are 48 SA2s and 30 PHAs. The SA2 level of denomination typically includes populations ranging from 3,000 to 25,000, with an average of approximately 10,000 people. These areas are designed to represent communities that interact socially and economically, serving as medium-sized general-purpose spatial units [24]. PHAs were developed by the Public Health Information Development Unit (PHIDU) at Torrens University, in collaboration with state and territory health agencies, to ensure data confidentiality and support the reporting of de-identified health information by merging smaller SA2s into a single PHA. In the case of the South Coast of NSW, four SA2s were merged to form a single PHA, three SA2s were merged to form a PHA twice, and two SA2s were merged to form nine PHAs, resulting in a total of 12 PHAs [25].

### Study design

An ecological analysis utilising secondary data from the Australian Bureau of Statistics (ABS), Population Health Information Development Unit, and mapping of the community food environments of the South Coast of NSW [20] was conducted to identify the association between community food environments and diet-related health outcomes.

### Mapping of the community food environment

The community food environment mapping was a desk-based cross-sectional study conducted between July 2023 and February 2024. We obtained the list of registered food outlets (including food outlet names and addresses) from the councils of each local government area. Food outlets listed in the registry were initially searched on Google Maps and their operational status verified. If an outlet was not found on Google Maps, the suburb name was added to the search; if it remained unidentifiable, it was marked as not currently operating. Additional food outlets not listed in the registry were identified through further Google Maps searches using various combinations of suburb names and outlet types using keywords including, “supermarket”, “grocery”, “general store”, “fruit shop”, “vegetable shop”, “green grocer”, “fruiterer”, “butchery”, “poultry”, “fish shop”, “fast food”, “takeaway shop”, “restaurant”, “café”, “PUB”, “alcohol shop”, “bottle shop”, “club”, “hotel”, “convenience store”, “bakery”, “delicatessen”, and “cake shop”.

Information on outlet type, menu, opening hours, service mode, and pictures was reviewed to identify each outlet’s main offerings and categorise them by type, based on predefined definitions (supplementary file 1). Thorough data cleaning was performed, removing duplicates and outlets not currently in operation. Home businesses, school canteens, temporary food stalls, sports complexes with limited hours, pharmacies, and other food outlets that do not open to the public, such as nursing homes, were excluded.

To assign a healthiness score, the type of food outlet was rated according to the Food Environment Score (FES), which uses a 20-point scoring system ranging from − 10 (least healthy) to + 10 (most healthy).

Food outlets were categorised into 18 categories based on the FES and then reclassified into 10 categories as follows for ease of reporting (Table 1).

**Table 1:** Categories of food outlets

New classification	FES category	Food environment score
Supermarket	Major supermarket, Minor supermarket and speciality food store (core)	+ 5
Fast-food outlet	Takeaway (franchise), Takeaway (local) and Speciality food store (extra)	-10 to -8
Restaurant/café	Restaurant/café (franchise) and Restaurant/café (local)	0
Bakery/cake shop	Bakery/cake shop and delicatessen	0
Convenience store	Convenience store and Service station convenience	-5 to -10
Fruiterer and greengrocer	Fruiterer and greengrocer	+ 10
Fish shop	Fish shop	+ 10
Butchery	Butchery	+ 9
Sandwich shop	Sandwich shop	+ 5
Alcohol outlets	Liquor selling stores and Pubs	-10 to -8

Based on the FES, food outlets were classified into three categories: healthy (FES + 5 to + 10); less healthy (FES − 4 to + 4); and unhealthy (FES − 10 to -5) [26]. Two independent raters performed the classification and healthiness score of food outlets, and differences in the rating were resolved by discussion. Various metrics of the community food environment were derived from the Community Food Environment Mapping dataset, as follows:

1. Density: The density of food outlets was calculated by dividing the number of food outlets by the total population at the SA2 level, expressed per 100,000 population. The population estimates were sourced from the Australian Bureau of Statistics 2021 census data [27].
2. Ratio: The ratio of unhealthy to healthy/less healthy food outlets was calculated for each SA2 and PHA level by dividing the number of unhealthy food outlets by the number of healthy and less healthy food outlets.
3. Relative Fast Food Availability: This metric indicates the relative availability of fast-food outlets compared to all food outlets and is calculated as the ratio of fast-food outlets to all food outlets at the SA2 and PHA levels.
4. The Modified Retail Food Environment Index (mRFEI): This metric shows the relative healthiness of food outlets within a geographic area. It was calculated at the SA2 and PHA level by dividing the number of healthy food outlets by all available food outlets (healthy, less healthy, and unhealthy) [28].
5. Relative Healthy Food Availability (RHFA): This metric measures the relative healthiness of food outlets within a geographic area, focusing on the healthiest and unhealthiest options. It was calculated by dividing the number of supermarkets, fruiterers, and greengrocers by the number of fast food outlets in each SA2 and PHA [29].

### Population-level diet-related health outcomes

The age-standardised prevalence of diabetes (all forms excluding gestational diabetes), heart disease, including heart attack and angina, kidney disease, and stroke at the SA2 level was calculated for individuals aged 25 years and older by obtaining the age-specific counts of diabetes, heart disease, kidney disease, and stroke from the Australian Bureau of Statistics (ABS) [24] census [30]. The age-standardised prevalence of high blood pressure, adults’ overweight, adults’ obesity, children’s overweight, children’s obesity, and self-rated health at the level of PHA was obtained from the Public Health Information Development Unit (PHIDU) of Torrens University, using their social health atlas [31] which also used the 2021 ABS census data. The Australian Bureau of Statistics used a single-item question for each long-term health condition to assess self-reported diagnoses in the 2021 census [32].

### Data analysis

The unit of analysis for this study was SA2 and PHA. The data was analysed using SPSS version 29.0. Mean/median with standard deviation (SD) and interquartile range (IQR) were used to summarise the health outcomes and community food environment metrics at the PHA and SA2 levels. Both the community food environment metrics and the prevalence of diet-related health outcomes were treated as continuous variables. Spearman’s correlation was used to identify the association between the community food environment and diet-related health outcomes. Linear regression was not conducted because the health outcome data and food environment metrics were collected at different time points. Due to the non-normal distribution of metrics of the community food environment, Spearman’s correlation coefficients were used to examine the association with health outcomes at both SA2 and PHA levels. Finally, the results were presented using tables, figures, and charts, and a P-value < 0.05 was used to determine statistical significance.

Graphical maps of the tertiles of the ratio of unhealthy to healthy/less healthy food outlets and age-standardised prevalence of diabetes at the SA2 level were created based on the ABS [24] census SA2 shapefile, using QGIS Desktop version 3.24.1. For mapping purposes, the ratio of unhealthy to healthy/less healthy food outlets and age-standardised prevalence of diabetes were ranked into three categories and presented as tertiles.

## Results

### Distribution of count and density of food outlets across the statistical area level 2 level

The restaurant/café category had the highest frequency and density per 100,000 population, followed by fast-food outlets. In contrast, fruiterers and greengrocers, as well as fishmongers, were lowest in both number and density at the SA2 level. The median count of restaurants and cafés across the SA2s was 17 (IQR: 7–24), with a median density of 13.8 per 10,000 population (IQR: 8.1–23.8). Fast-food outlets had a median count of 12.5 (IQR: 6–20) and a median density of 10.6 per 10,000 population (IQR: 8–15). Supermarkets had a median count of 3.5 outlets (IQR: 2–7), with a median density of 3.3 per 100,000 population (IQR: 2.4–5.4) (Table 2).

**Table 2:** The median and interquartile range of the number and density of types of food outlets at the SA2 level (*N* = 42) of the South Coast of NSW, Australia

Metrics	Number		Density per 10,000 population	
	Median	Inter Quartile Range (IQR)	Median	IQR
Supermarket	3.5	(2–7)	3.3	(2.4–5.4)
Fast-food outlet	12.5	(6–20)	10.6	(8.1–14.9)
Restaurants/cafes	17	(7–24)	13.8	(8.1–23.8)
Convenience stores	4	(2–5)	3.0	(1.8–5.4)
Number of alcohol outlets	5	(3–8)	5.1	(2.6–8.4)
Bakery/cake shop	2.5	(1–4)	2.5	(1.1–3.8)
Fruiterer and greengrocer	0	(0–1)	0	(0.0-0.7)
Fish shop	0	(0–1)	0	(0.0-0.5)
Butcheries	1	(1–2)	1.2	(1.2–2.2)
Sandwich shop	1	(0–2)	0.5	(0.0-1.2)
Healthy food outlets^a^	6	(3–9)	5.6	(4.2–10.2)
Less healthy food outlets^b^	19.5	(9–28)	16.4	(9.8–27.7)
Unhealthy food outlets^c^	21.5	(12–33)	19.5	(13.7–29.3)

^a^Healthy food outlets: supermarkets, fruiterers and greengrocers, fishmongers, butchery, and sandwich shops

^b^Less healthy food outlets: restaurants/cafés and baker’s/cake shops

^c^Unhealthy food outlets: convenience stores, alcohol outlets, and fast-food outlets

Unhealthy food outlets were highest in frequency, as well as density per 100,000 population in the SA2. In contrast, healthy food outlets were lowest in frequency and density. The median count of unhealthy food outlets per SA2 was 21.5 (IQR: 12-32.5), with a median density of 19.5 per 10,000 population (IQR: 13.7–29.3). The median count of less healthy food outlets was 19.5 (IQR: 9–28) while healthy food outlets had a median count of 7.5 (IQR: 4–11) and a median density of 5.6 per 10,000 population (IQR: 4–10) (Table 2).

### Relative metrics of healthy and unhealthy food outlets across the statistical area level 2 level

Median (IQR) scores are shown in Table 3 for the Modified Retail Food Environment Index (nMRFEI), the Relative Healthy Food Availability (RHFA), the Relative Fast-Food Outlets Availability (RFFA), and the Relative Healthy Food Availability (RHFA). The median ratio of unhealthy to healthy/less healthy food outlets was 0.92 (IQR: 0.66–1.24) (Table 3).

**Table 3:** The median and interquartile range of the relative metrics of community food environments at the SA2 level (*N* = 42) of the South Coast of NSW, Australia

Metrics	Median	Interquartile range (IQR)
nMRFEI^a^	0.14	(0.11–0.18)
RHFA^b^	0.36	(0.24–0.66)
RFFA^c^	0.27	(0.19–0.34)
Ratio of unhealthy to healthy/less healthy food outlets	0.92	(0.66–1.24)

^a^*mRFEI* Modified Retail Food Environment Index

^b^*RHFA* Relative Healthy Food Availability

^c^*RFFA* Relative Fast-Food Availability

### Summary of diet-related long-term health conditions according to statistical area level 2 (SA2)

Among individuals aged 25 years and older at the SA2 level, diabetes demonstrated the highest age-standardised prevalence, with a mean (SD) of 6.28% (1.63) and a range from 3,49% to 9.69%, followed by heart disease with a mean (SD) of 5.58% (0.65). In contrast, stroke exhibited the lowest prevalence, with a mean (SD) of 1.38% (0.29), ranging from 0.96% to 1.97% (Table 4).

**Table 4:** The descriptive statistics of age-standardised prevalence of diet-related long-term health conditions at the SA2 level (*N* = 42) of the South Coast of NSW, Australia

Health conditions	Mean age-standardised prevalence (%)	Standard deviation (SD)	Minimum	Maximum
Diabetes	6.28	1.63	3.49	9.69
Heart disease	5.58	0.65	4.42	7.30
Kidney disease	1.62	0.37	0.88	2.7
Stroke	1.38	0.29	0.96	1.97

### Summary of diet-related health outcomes according to the population health area (PHA)

At the PHA level, overweight (not obese) had the highest age-standardised prevalence among individuals aged 15 years and above, with a mean (SD) of 34.3% (0.50), ranging from 33.2% to 35.20%, followed by obesity with a mean (SD) of 32.60% (4.42), while poor or fair self-assessed health had the lowest prevalence, with a mean (SD) of 14.65% (2.64), ranging from 9.60% to 19.50% (Table 5).

**Table 5:** The descriptive statistics of age-standardised prevalence of diet-related long-term health conditions at the PHA level (*N* = 28) of the South Coast of NSW, Australia

Health conditions	Mean age-standardised prevalence (%)	Standard deviation (SD)	Minimum	Maximum
Poor or fair self-assessed health	14.65	2.64	9.60	19.50
High blood pressure	24.53	1.02	22.30	27.70
Overweight (not obese)	34.30	0.50	33.20	35.20
Obesity	32.60	4.42	24.60	40.30
Children overweight	18.11	0.39	17.20	18.90
Children obesity	7.94	1.54	5.10	10.30

Among children aged 2–17 years at the level of PHA, the age-standardised prevalence of overweight was 18.11% (0.39), ranging from 17.20% to 18.90%. For obesity, the prevalence was 7.94% (1.54), with a range of 5.10% to 10.30% (Table 5).

### Association between metrics of community food environment and diet-related health outcomes

Among individuals aged 25 years and older at the SA2 level, the age-standardised prevalence of diabetes was positively associated with the ratio of unhealthy to healthy/less healthy outlets (ρ = 0.48, *P* = 0.001) and the Relative Fast Food Availability (RFFA) (ρ = 0.60, *P* < 0.001). Likewise, positive associations were observed between the age-standardised prevalence of stroke and the ratio of unhealthy to healthy/less healthy outlets (ρ = 0.32, *P* = 0.042) as well as with RFFA (ρ = 0.43, *P* = 0.005). Similarly, the age-standardised prevalence of kidney disease was also positively associated with the ratio of unhealthy to healthy/less healthy outlets (ρ = 0.42, *P* = 0.006) (Table 6).

**Table 6:**
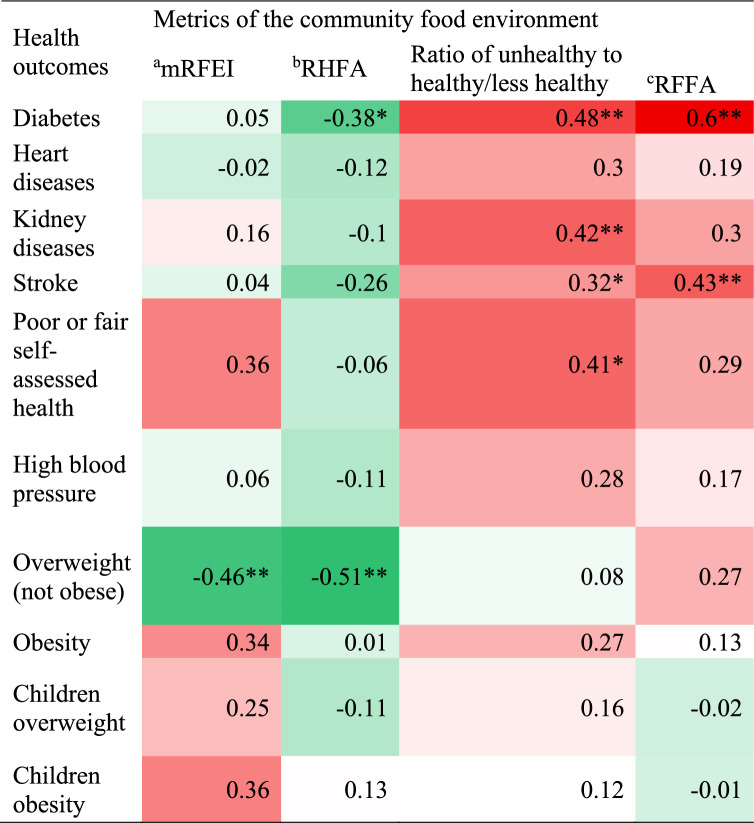
The correlation between metrics of community food environment and diet-related health outcomes according to the SA2 and PHA levels of the South Coast of NSW, Australia

^a^*mRFEI* Modified Retail Food Environment Index Red: Strong positive association

^b^*RHFA* Relative Healthy Food Availability White: No association

^c^*RFFA* Relative Fast-Food Availability Green: Strong negative association

*Significant at *P* < 0.05; **Significant at *P* < 0.01

Among individuals aged 15 years and older at the PHA level, a positive association was found between the ratio of unhealthy to healthy/less healthy food outlets and the age-standardised prevalence of poor or fair self-assessed health (ρ = 0.41, *P* = 0.029) (Table 6).

The age-standardised prevalence of diabetes among individuals aged 25 years and older at the SA2 level was inversely associated with Relative Healthy Food Availability (RHFA) (ρ = -0.38, *P* = 0.012). Likewise, among individuals aged 18 years and older at the PHA level, the age-standardised prevalence of overweight was inversely associated with the Modified Food Environment Index (mRFEI) (ρ = -0.46, *P* = 0.013) and the RHFA (ρ = -0.51, *P* = 0.005) (Table 6).

### Mapping of the ratio of unhealthy to healthy/less healthy outlets and age-standardised prevalence of diabetes at the statistical area level 2

**Fig. 1:**
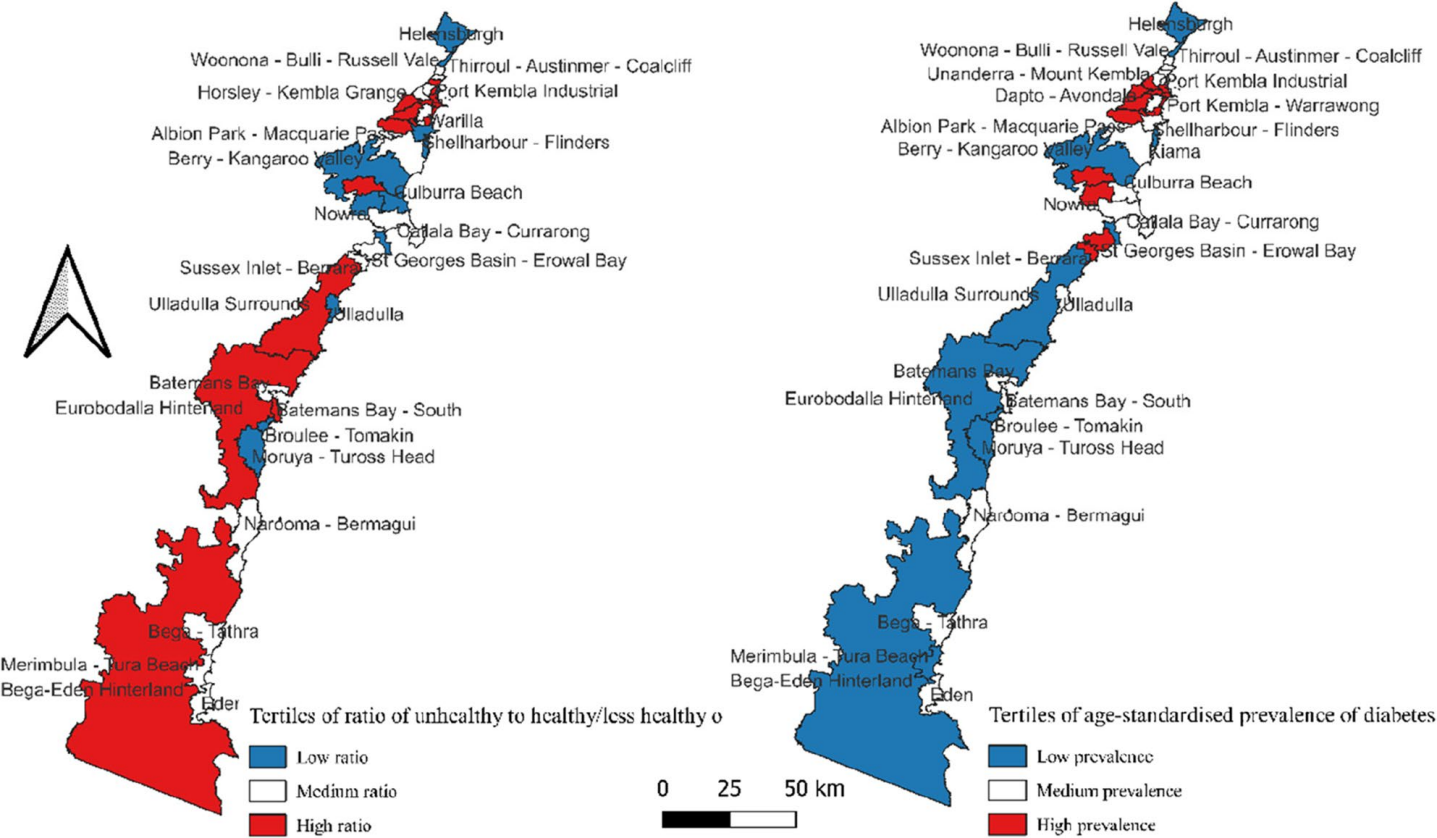
Tertiles of the ratio of unhealthy to healthy/less healthy outlets (map 1) and the age-standardised prevalence of diabetes (map 2) according to the SA2 level of the South Coast of NSW, Australia

Tertiles of the ratio of unhealthy to healthy/less healthy outlets are mapped according to SA2 level, alongside tertiles of diabetes prevalence for the same geographic denominations (Fig. 1). Notably, several SA2s in the Northern part of the region (Port Kembla-Warrawong, Albion Park-Macquarie Pass, Albion Park Rail, Horsley-Kembla Grange, Dapto-Avondale, Berkeley - Lake Heights – Cringila, Warilla, North Nowra-Bomaderry appear in the highest tertile of the ratio of unhealthy to healthy/less healthy outlets and also have the highest age-standardised prevalence of diabetes, suggesting a potential spatial correlation between regional-level metrics of community food environment and diabetes.

## Discussion

To the best of our knowledge, this is the first study in a large regional area of Australia to explore the ecological associations between several objective metrics of the community food environment and population-level health outcomes, including diabetes, heart disease, kidney disease, stroke, high blood pressure, overweight, obesity, and poor or fair self-assessed health. Across the South Coast of New South Wales, the community food environment is characterised by a high density of unhealthy food outlets (particularly restaurants, fast-food outlets and convenience stores) with limited access to fresh food retailers, such as fruit and vegetable shops and fishmongers. Understanding these area-level patterns is an important step in identifying which food environment metrics are most relevant for future individual-level research and which health outcomes should be prioritised for further investigation. While the ecological nature of the study precludes individual-level inference, our findings are consistent with emerging international evidence that neighbourhood food environments may be associated with area-level variation in health outcomes [15, 16, 33].

Evidence from regional Australia shows that food environments differ from those in metropolitan areas in terms of outlet distribution, accessibility, and their interactions with broader structural issues such as transport and supply chains. These factors may result in an increased reliance on convenience foods and unhealthy outlets [4–6, 34]. Our finding of an unhealthy food outlet dominance in the South Coast region of NSW, Australia, supports this. In particular, areas with a higher proportion of fast-food outlets and convenience stores, relative to other healthier options, were associated with an increased prevalence of diabetes, stroke, kidney disease, and poor self-rated health. Our study extends the findings from previous reviews that have focused primarily on overweight or obesity alone as a health outcome [15, 35]. Specifically, our study aligns with the growing evidence [16, 30–32] that a high proportion of fast-food outlets is associated with a higher prevalence of diabetes and stroke. This relationship may be explained by the fact that unhealthy food outlets, including fast-food outlets and convenience stores, are a key source of nutrient-poor and energy-dense foods in the community, which contribute to unhealthy dietary patterns [36, 37]. Further, these outlets are disproportionately located in socioeconomically disadvantaged locations [34, 38, 39] including within regional and rural settings [40–42]. This pattern was confirmed in our prior mapping of this region [20], and is supported by a scoping review by Madlala et al. [43], which demonstrated that the community food environment has a significant influence on food choices among adults in regional and low socioeconomic communities [43]. We thus propose that metrics based on unhealthy food outlets may serve as a sensitive and meaningful measure of the community food environment when examining its association with diet-related health outcomes, particularly in regional and low socioeconomic areas.

Conversely, supermarkets and greengrocers are key sources of affordable, nutrient-dense foods. Their presence within a community food environment has been shown to improve both physical access and purchasing opportunities for healthier options, which can lead to improved dietary quality and reduced risk of chronic diseases at the population level, supported by our study [44–46]. Similarly, Thorpe et al. [47] and Kanchi et al. [48] reported that the relative availability of supermarkets in the neighbourhood reduces the risk of type 2 diabetes [47, 48]. Another longitudinal study reported that healthy food availability, along with physical activity resources in the neighbourhood, is associated with a reduced incidence of type 2 diabetes [49]. Regarding overweight and/or obesity, a number of systematic reviews have reported null associations with food environment metrics [13, 14, 50]. This may be due to the complex and multifactorial causes of obesity, which include not only food access but also individual behaviours, cultural norms, sedentary lifestyles, stress, and genetics [51]. However, the most recent review showed that proximity to supermarkets and the density of fruit and vegetable shops were inversely associated with obesity [15]. A higher density of healthy food outlets and a more positive perception of the food environment have been shown to be associated with higher intakes of fruits and vegetables [52, 53], which may explain this relationship.

Our ecological study findings join the growing body of research linking community food environments to health outcomes, with important implications for local policy and practice. Effective policy responses and public health initiatives can enhance the healthfulness of community food environments and reduce diet-related non-communicable diseases at the local level [54]. Interventions aimed at improving food environments in community settings, such as schools [55–58], workplaces [59–61], and neighbourhoods [62–64], are promising but remain underutilised in Australia. Promising intervention strategies include zoning regulations to limit unhealthy food outlets, particularly fast-food outlets near schools [65], the establishment of healthy food outlets [66], implementation of healthier food procurement policies [67], and the provision of nutrition education programs that are targeted at behaviour change [68]. At the policy level, food labelling, restrictions on unhealthy food marketing, and fiscal policies such as taxes on sugar-sweetened beverages have shown promise internationally [69]. These types of interventions are particularly essential for promoting long-term health equity in regional and rural contexts in Australia, where food access and diet-related health disparities are pronounced. Monitoring of food environment policies at both the local government, state, and national levels can be conducted using validated instruments such as the Food-EPI tool; however, there are no systems or support in place for this to occur routinely [70, 71]. Future work should examine the design, implementation, and equity impact of food environment interventions tailored to regional contexts.

This study has several strengths, including the use of multiple objective measures of the community food environment and the inclusion of various diet-related health outcomes, expressed as age-standardised prevalence. However, there are some limitations to consider. First, this study focuses on food availability and overlooks other important dimensions of the food environment, including access, affordability, and promotion, which should be investigated separately. Also, the study used three different datasets collected at different time points. As the prevalence of diet-related health outcomes and food environment may change over time, this may influence the observed associations, which were not considered in the study. In addition, the study relied on secondary, aggregated data, which limited the level of clinical detail available to characterise diet-related health outcomes. Health conditions were based on self-reported diagnosis of long-term conditions, assessed using a single-item self-reported survey question. As a result, it was not possible to disaggregate conditions by subtype or more detailed clinical characteristics. Our study is also influenced by the ecological design, including potential bias related to the ecological fallacy, whereby associations identified at the community level may not be reflective of those at the individual level, and is unable to distinguish whether the associations relate to geographic variations of the community food environment or to individual characteristics of residents living in different areas. The study used correlation as a measure of association, which cannot account for potential confounding. Future studies should be conducted that collect individual-level data on both objective and perceived community food environments, including access and affordability, and link these to dietary intake and relevant health outcomes.

## Conclusion

This ecological study provides area-level evidence from a large regional area of Australia that a high relative availability of unhealthy food outlets was associated with an increased age-standardised prevalence of diabetes, kidney disease, stroke, and poor self-rated health, while a greater presence of healthy food outlets was associated with a lower prevalence of overweight and diabetes. The findings highlight important spatial patterns that suggest the community food environment plays a role in shaping population-level health outcomes. These results join a growing body of literature that supports the need for policies that create healthier community food environments as part of broader strategies to reduce the overall burden of diet-related diseases. The findings also support the need for further research, particularly at the individual level, to confirm these associations and inform policy and public health interventions.

## Data Availability

The data are available on request from the corresponding author.

## Abbreviations

ABDS: Australian Burden of Disease Study
ABS: Australian Bureau of Statistics
CALD: Culturally and Linguistically Diverse
FES: Food Environment Score
IQR: Inter Quartile Range
LGA: Local Government Area
mRFEI: Modified Retail Food Environment Index
NSW: New South Wales
PHA: Population Health Area
PHIDU: Public Health Information Development Unit
RHFA: Relative Healthy Food Availability
RFFA: Relative Fast-Food Availability
SA2: Statistical Area Level two
SD: Standard Deviation
